# Effect of *trans*-Cinnamaldehyde on Adhesion and Other Virulence Factors of Methicillin-Resistant *Staphylococcus aureus*

**DOI:** 10.3390/pathogens15030271

**Published:** 2026-03-03

**Authors:** Barbara Kot, Kamila Wierzchowska, Agata Grużewska, Elżbieta Anna Trafny, Małgorzata Stępińska, Małgorzata Witeska

**Affiliations:** 1 Institute of Biological Sciences, Faculty of Exact and Natural Sciences, University of Siedlce, 14 Bolesława Prusa Str., 08-110 Siedlce, Poland; kamila.wierzchowska@uws.edu.pl; 2 Institute of Agriculture and Horticulture, Faculty of Agrobioengineering and Animal Husbandry, University of Siedlce, 12 Bolesława Prusa Str., 08-110 Siedlce, Poland; agata.gruzewska@gmail.com; 3 Biomedical Engineering Centre, Institute of Optoelectronics, Military University of Technology, Kaliskiego 2, 00-908 Warsaw, Poland; elzbieta.trafny@wat.edu.pl (E.A.T.); malgorzata.stepinska@wat.edu.pl (M.S.); 4 Department of Animal Environment Biology, Institute of Animal Science, Warsaw University of Life Sciences (SGGW), Ciszewskiego 8, 02-786 Warsaw, Poland; malgorzata_witeska@sggw.edu.pl

**Keywords:** MRSA, *trans*-cinnamaldehyde, adhesion, inhibition, virulence

## Abstract

Methicillin-resistant *Staphylococcus aureus* (MRSA) produces virulence factors and causes hard-to-treat infections. This study aimed to evaluate the effect of *trans*-cinnamaldehyde (TC) on the selected virulence factors of MRSA: adhesion to host plasma and extracellular matrix proteins, protease, DNase and esterase production, and hemolytic activity. Our results showed that TC at ½ MBIC (Minimum Biofilm Inhibition Concentration) of 240 µg/mL or 60 µg/mL, depending on the isolate, significantly reduced MRSA adhesion. Inhibition varied between isolates, ranging from 26.1% to 41.3% (fibrinogen), 18.2% to 34.9% (elastin), 26.5% to 32.4% (laminin), and 17.1% to 30.5% (collagen). TC at ½ MIC (Minimum Inhibitory Concentration) of 30 µg/mL also significantly inhibited MRSA enzyme production, and reduced hemolytic activity (by 80.0–83.1%, depending on the isolate). TC may be an alternative to antibiotics for combating infections caused by *S. aureus*, as it not only reduces bacterial survival in the host but also reduces *S. aureus* virulence at subinhibitory concentrations. TC at higher concentrations exhibits cytotoxicity in human fibroblasts, limiting its topical use. Therefore, to exploit TC’s antibacterial potential, it is necessary to identify substances that act synergistically with it, enabling reduced effective doses.

## 1. Introduction

*Staphylococcus aureus* rapidly acquires antibiotic resistance and is the most common cause of nosocomial infections. Methicillin-resistant *S*. *aureus* (MRSA) leads to infections that are difficult to treat and poses a growing threat to human health. MRSA is considered a high-priority antibiotic-resistant pathogen by the WHO. WHO data (2025) indicate that MRSA accounted for 27.1% of global bloodstream infections, with the highest proportion observed in the Eastern Mediterranean Region at 50.3% [1]. In 2024, the estimated incidence of MRSA bloodstream infections in the European Union was 4.48 cases per 100,000 inhabitants, with national rates ranging from 0.55 to 13.63 cases per 100,000 inhabitants [2]. MRSA gains resistance via the staphylococcal cassette chromosome mec carrying the *mecA* gene, that encodes the low-affinity penicillin-binding protein 2a. This process confers resistance to β-lactam antibiotics [3]. In addition to β-lactam antibiotics, MRSA frequently exhibits resistance to other antibiotic classes, including fluoroquinolones, aminoglycosides, tetracyclines, macrolides, and chloramphenicol [4]. The course and severity of host infection with *S. aureus* depend on the virulence factors it produces. Biofilm formation is a key factor in the persistence of infections throughout human tissues. The progression of *S. aureus* infections also relies on other virulence factors, including adhesins, degradative enzymes, and toxins. The scarcity of effective treatments for MRSA infections leads to more lives lost and drives up healthcare expenses. Using plant metabolites as antimicrobial agents offers a promising alternative to antibiotics. *trans*-cinnamaldehyde (TC) is a phenolic compound with antibacterial activity. The antibacterial activity of TC depends on the presence of the acrolein group (α,β-unsaturated carbonyl moiety), which enables covalent interactions with thiol, amino, and hydroxyl groups present in microbial proteins and enzymes. These reactions can lead to enzyme inactivation, disruption of essential metabolic pathways, and impairment of cell viability [5,6]. TC is an antimicrobial agent characterized by low toxicity at minimal doses and is recognized as safe for use in foods by the US Food and Drug Administration (FDA) [7]. Derived from cinnamon bark, this bioactive compound demonstrates anticancer, antifungal, and antibacterial properties. Its antimicrobial activity results from inhibiting ATPase activity, disrupting cell wall biosynthesis, and altering cell membrane structure and integrity, thereby inhibiting bacterial growth [8]. In *S. aureus,* TC induces cell wall destruction and alters cell membrane permeability, leading to the release of proteins, alkaline phosphatase, potassium ions, and other intracellular components [9].

Many authors have assessed the antimicrobial activity of cinnamaldehyde by determining minimum inhibitory concentrations (MICs) for bacterial growth and biofilm development [10]. However, data on the effects of cinnamaldehyde on the production of bacterial virulence factors are limited, especially when tested at subinhibitory concentrations. The effect of TC on specific MRSA virulence factors is poorly understood. Only a few studies have assessed the effect of TC on the hemolytic activity of *S. aureus* [11,12] and the expression of genes encoding adhesion proteins [13]. To date, no studies have examined the effects of TC on the production of other virulence factors, such as proteases, DNase, and lipases, or on the adhesion of *S. aureus* to host proteins. In our study, we analysed the effect of TC on the adhesion of MRSA biofilm cells to host plasma proteins (fibrinogen) and to the extracellular matrix proteins (elastin, laminin, and collagen). Host tissue damage by *S. aureus* is the result of the action of enzymes and toxins produced and released by bacterial cells. This study aimed to evaluate the effect of subinhibitory concentrations of TC on adhesion to host proteins, enzyme production, hemolytic activity, in MRSA clinical isolates, and cytotoxicity to human fibroblasts.

## 2. Materials and Methods

### 2.1. Bacterial Isolates

MRSA isolates utilized in this study were collected in 2017 from patients hospitalized in Siedlce, Poland. These isolates were obtained as part of routine diagnostic microbiology and originated from wound, nasal, and anal samples. Sterile swabs were used for sample collection, which were then inoculated onto 5% sheep blood agar (Graso Biotech, Starogard Gdański, Poland). The blood agar plates were incubated aerobically at 37 °C for 24 h. Isolates were identified as *S. aureus* using Gram staining, catalase, and tube coagulase tests. PCR targeting the *nuc* gene, which encodes a thermostable nuclease specific to *S. aureus*, confirmed species identity [4]. The methods for identification of the *mecA* gene, responsible for resistance to β-lactam antibiotics, and the *nuc* gene have been described previously. Monoplex PCR targeting the *nuc* and *mecA* genes was performed in a 25 µL reaction containing 2.5 µL DNA template, 1× PCR buffer, 0.2 mM each dNTP (Fermentas, Vilnius, Lithuania), 150 nM specific primers, and 1 U RedTag Genomic DNA polymerase (Sigma Aldrich, Steinheim, Germany). The amplification protocol consisted of an initial denaturation at 94 °C for 4 min, followed by 35 cycles of 94 °C for 30 s, 55 °C for 30 s, and 72 °C for 30 s, with a final extension at 72 °C for 5 min [4]. Methods for determining the minimum inhibitory concentration (MIC) and minimum biofilm inhibitory concentration (MBIC) of TC (Sigma-Aldrich, Steinheim, Germany, lot no. MKBW8907V) have been described previously [13]. MRSA isolates analyzed in this study are listed in Table 1. To assess the effect of TC on MRSA virulence factors, three different groups of isolates with specific virulence traits were selected and used in three different assays (adhesion, hemolysis and enzyme production assays).

### 2.2. Evaluation of Adhesion of MRSA Isolates Treated with TC to Proteins of the Host Plasma and Extracellular Matrix

In this study two isolates forming strong biofilm (27887, 30216) and one isolate weakly adhering to polystyrene (1037) were used [14]. Wells of tissue culture polystyrene 96-well plate (Nunclon, Roskilde, Denmark) were coated with fibrinogen from human plasma, laminin from human fibroblasts, elastin from human skin and human type I collagen (all from Sigma-Aldrich) by filling with 10 μg/mL protein solution in sterile phosphate-buffered saline—PBS (pH 7.4) (Graso Biotech, Starogard Gdański, Poland) (or 0.2% acetic acid for collagen). The plates with fibrinogen, laminin and elastin were previously incubated for 2 h at 37 °C and then incubated overnight at 4 °C. Plates with collagen were incubated overnight at 37 °C. Afterwards, remaining proteins were removed with a pipette. To assess adhesion of bacterial cells to proteins, bacterial cells derived from biofilm were used.

MRSA isolates were cultured in tryptic soy broth (TSB; BBL, Becton Dickinson, Sparks, Sparks Glencoe, MD, USA) supplemented with 0.5% glucose at 37 °C for 18 h. Cultures were then inoculated onto tryptic soy agar (TSA) containing 0.5% glucose and incubated at 37 °C for an additional 18 h. Bacterial cells from each isolate were suspended in phosphate-buffered saline (PBS; pH 7.4) to achieve an optical density at 565 nm (OD565) of 3.2. These suspensions were diluted with TSB containing 0.5% glucose and tetracycline (TC) at final concentrations of one-half minimum biofilm inhibitory concentration (MBIC; 240 µg/mL for isolate 277887 and 60 µg/mL for the remaining isolates) to prepare cell suspensions containing approximately 1 × 10^8^ CFU/mL. For biofilm formation, 1 mL of each bacterial cell suspension in TC was transferred to wells of a tissue culture polystyrene 24-well plate (Nunc, Roskilde, Denmark) in 10 replicates and incubated without agitation for 12 h at 37 °C. Bacterial growth controls were prepared in wells containing bacterial cell suspensions in TSB with 0.5% glucose. Following incubation, the medium was removed, and non-adherent bacterial cells were eliminated by washing the biofilms twice with 250 µL of sterile PBS. Adherent bacterial cells were then scraped using a pipette and resuspended in an appropriate volume of PBS to obtain suspensions containing approximately 1 × 10^8^ CFU/mL. Subsequently, 100 µL of each bacterial suspension was added to wells coated with the relevant protein and incubated for 2 h at 37 °C. After incubation, bacterial suspensions were removed, and wells were washed once with PBS. Bacterial cell adhesion was assessed using the resazurin microtiter-plate assay. For this assay, 190 µL of TSB containing 0.5% glucose and 10 µL of resazurin were added to each well, and the microplates were incubated for 3 h in the dark at 37 °C. Absorbance was measured at 492 nm using a microplate reader(Apollo LB913, Berthold Technologies, Bad Wildbad, Germany). Each assay was performed in triplicate, and the results were averaged.

Adhesion inhibition (%) of MRSA isolates to the investigated proteins was evaluated by comparison of the absorbance value for adhering bacterial cells treated with TC at ½ MBIC to the absorbance value of the control. Adhesion inhibition was calculated according to the formula: Adhesion inhibition (%) = [(Control OD_492nm_ − Treated OD_492nm_)/Control OD_492nm_] × 100.

### 2.3. Assessment of Virulence Factor Production by MRSA Treated with TC

The MRSA isolates that produced proteases, DNase and esterases, collected from the nose (292911), anus (1037) and wound (1559), were used to study the effect of TC at ½ MIC (30 µg/mL) on the production of these enzymes.

#### 2.3.1. Extracellular Protease Production

The ability of TC-treated and untreated MRSA isolates to produce proteases was determined by plating the bacterial culture as a band streak on Tryptic-Soy Agar (TSA; BBL, Becton Dickinson, Sparks, Md., Franklin Lakes, NJ, USA) supplemented with 3% (*w*/*v*) bovine gelatine (Sigma Aldrich) and TC or without TC. MRSA isolates were incubated at 35 ± 2 °C. After 48 h of incubation, 1 N hydrochloric acid (Sigma Aldrich) was added to the surface of the plates. The presence of a clear zone near the bacterial growth, resulting from exoprotease activity, was confirmed, and its size was measured [15]. The mean values of three independent experiments were calculated.

#### 2.3.2. Extracellular DNase Production

The tested MRSA isolates were inoculated onto DNase Test Lab-Agar (BioMaxima, Lublin, Poland) with and without TC as a band streak technique. The plates were incubated for 48 h at 35 ± 2 °C. After that, 1 N hydrochloric acid (Sigma Aldrich) was added to the surface of the plates. After 5 min, in the presence of hydrochloric acid, the reaction with DNA in the medium formed a hazy precipitation, while the isolates producing deoxyribonuclease were surrounded by a clear zone containing fractions of soluble nucleotides from the DNA degradation that were not precipitated by the hydrochloric acid. Zone sizes were measured, and the mean values across the three independent experiments were calculated.

#### 2.3.3. Esterase Production

For evaluation of the esterase production in the presence of TC, the TSA plates containing 0.1% Tween 80 (Sigma-Aldrich), calcium chloride (Sigma-Aldrich) and TC were streaked with a fresh culture of MRSA isolates (24 h at 37 °C in Tryptic-Soy Broth) and incubated at 35 ± 2 °C for 48 h. Growth of isolates on plates without TC was the control. The reading was carried out after this period, according to the protocol proposed by Chapin and Murray [16]. Zone sizes were measured, and the mean values across the three independent experiments were calculated.

#### 2.3.4. Hemolysis Assay

Nasal (1059) and wound (1530, 2245) MRSA isolates with hemolytic activity were selected to evaluate the effect of TC at half the minimum inhibitory concentration (30 µg/mL) on hemolysis. Supernatants were collected from MRSA cultures in tryptic soy broth (TSB; BBL, Becton, Dickinson) with and without TC after 24 h of incubation at 37 °C. Sheep erythrocytes were isolated by centrifuging blood at 3000× *g* for 5 min, followed by plasma removal and three washes with phosphate-buffered saline (PBS) before dilution in PBS. Hemolysis was measured by incubating 1 mL of supernatant with 1 mL of sheep red blood cells (3% *v*/*v* in PBS) for 2 h at 37 °C. After incubation, samples were centrifuged at 12,000× *g* for 5 min, and supernatants were transferred to microplate wells for optical density measurement at 492 nm (Apollo LB913, Berthold Technologies, Bad Wildbad, Germany). Each assay was performed in triplicate, and the results were averaged. Hemolysis reduction was calculated as follows: Hemolysis reduction (%) = [(Control OD_492nm_ − Treated OD_492nm_)/Control OD_492nm_] × 100 [17].

### 2.4. Cytotoxicity Study of TC on Human Fibroblast Cell Culture In Vitro

Human fibroblast cells from the skin of an adherent primary line (LONZA, CC-2511 NHDF) were studied. The cell line showed adherent growth under in vitro culture conditions and was biological material with a risk level 1 (BSL-1). Cell culture was carried out in RPMI 1640 medium with stable glutamine (ECM 2001L, Euroclone S.p.A., Pero, Italy) and supplements required for fibroblast growth: 10% FBS (Fetal Bovine Serum) (A5256701, Gibco, Thermo Fisher Scientific, Grand Island, NY, USA) with the addition of a mixture of antibiotics (Pen/Strep/Fungizone, 17-745E, Lonza, Basel, Switzerland). The cell suspension, stored in liquid nitrogen, was quickly thawed in a water bath at 37 °C for 2 min and transferred to a 15 mL Falcon tube. After centrifugation of the cell suspension at 225× *g* for 5 min at room temperature and resuspension of the cell pellet in 1–5 mL of new culture medium, the number of cells in suspension was determined. Approximately 2–3 × 10^3^ cells/mL were cultured in a 75 mL culture dish for 5 days to 80–90% confluence in a tissue culture incubator (INCOmed 153, Memmert, Schwabach, Germany) in an atmosphere of 5% CO_2_ at 37 °C and humidity of 90%. The growth of the cultures was monitored by inverted microscope observations (Nikon Ts2R-FL, Tokyo, Japan). The culture medium was changed after the first and fourth days of cell cultivation.

For the final experiment, cells were passaged on the 5th day of culture from culture bottles into 96-well plates for cytotoxicity testing of TC. Passage of the fibroblast culture was performed after detaching the cells from the bottom of the culture vessel. Cell monolayer was treated with a trypsin solution (Lonza CC-5012) from the Reagent Pack Subculture kit (Lonza CC-5034) at a concentration of 0.025% (*w*/*v*) in a volume depending on the size of the culture vessel (2–3 mL per 75 mL bottle), incubating at room temperature for up to 5 min. Subsequently, the total number and percentage of live and dead cells in the cell suspension were calculated. An amount of 20 µL of cell suspension was stained with 20 µL of trypan blue solution (Invitrogen) at a concentration of 0.4% (*w*/*v*), and then 10 µL of the stained cell sample was applied to the chamber in the Countess Cell Counting Chamber Slides dedicated to the Countess apparatus (Life Technologies, Carlsbad, CA, USA). The wells of a 96-well microplate were seeded with a cell suspension of 100 µL at a density of 2 × 10^5^ cells/mL. Fibroblasts were cultured for 24 h in an incubator and then exposed to TC pre-dissolved in DMSO and diluted in the medium. Serial 2-fold dilutions of TC were made in the medium, ranging from 15,360 µg/mL to 0.469 µg/mL. After 24 consecutive hours of incubation of the confluent fibroblasts with TC, a cytotoxicity test was performed using PrestoBlue, Cell Viability Reagent (PB, Molecular Probes, Invitrogen, CA, USA). The healthy control consisted of wells coated with cells in pure culture medium (2-day culture), and the DMSO interaction control consisted of wells coated with cells in medium containing 2-fold dilutions of DMSO alone. Standard fibroblast culture medium with the addition of PrestoBlue reagent was also used as a so-called blank control on clean microplate wells not coated with cells. The study included five independent experiments for each concentration of the test substance, with each replicate in two (total N = 10 per dilution). After 24 h, cell metabolic activity was assessed using PrestoBlue reagent. The reagent was prepared according to the manufacturer’s instructions. After removing the medium above the cells and gently washing the culture with pure DPBS (ATCC), a mixture of 90 μL of medium and 10 μL of PrestoBlue reagent was added to each well of the plate. The cells were incubated with the reagent for 2 h in an incubator (INCOmed 153, Memmert, Schwabach, Germany) under 5% CO_2_ and 90% humidity at 37 °C. Fluorescence was then read in a multi-reader (ClarioStar, BMG Labtech, Ortenberg, Germany). For each dilution of the tested mixture, the percentage change in fluorescence compared to the blank was calculated. The PrestoBlue Cell Viability Reagent used in these studies contains resazurin. Resazurin is blue and non-fluorescent. In live cells, it is reduced in the respiratory chain to resorufin, a red and fluorescent compound. Fluorescence intensity readings were taken from the top in endpoint mode with an excitation wavelength of 545 ± 20 nm and an emission wavelength of 600 ± 40 nm. The dichroic mirror was automatically set to 498.8 nm, and the gain was corrected to 1000.

### 2.5. Statistical Analysis

Results are shown as mean plus or minus standard deviation. All statistical analyses were carried out with STATISTICA 12 from StatSoft in Cracow, Poland. The Shapiro–Wilk test verified that all values followed a normal distribution. To assess the significance of variables in MRSA isolates, analysis of variance was performed, followed by Tukey’s honestly significant difference test.

## 3. Results

### 3.1. Inhibitory Effect of TC on Adhesion of MRSA Biofilm Cells to Proteins Present in the Host Plasma and Extracellular Matrix

**Figure 1:**
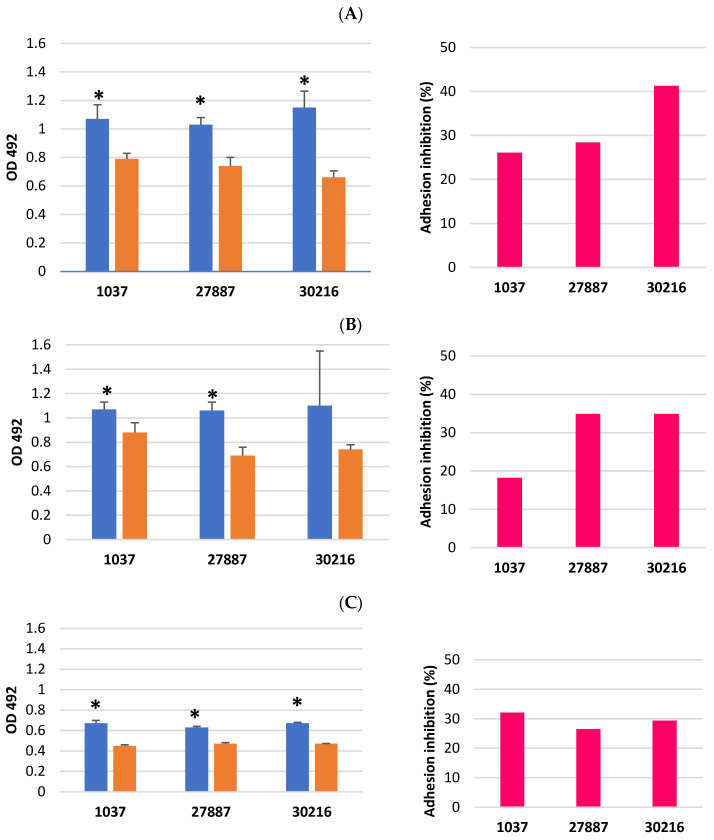

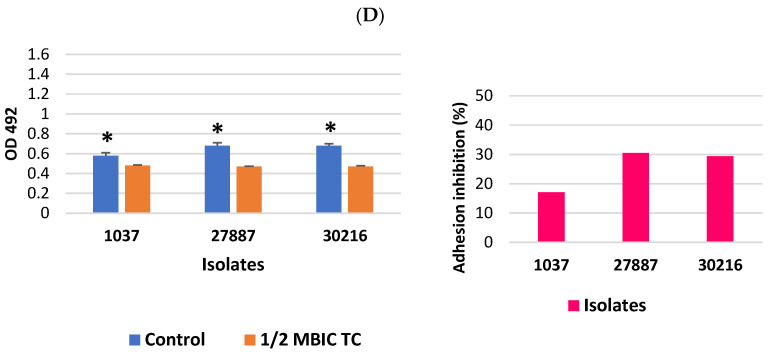
The effects of *trans*-cinnamaldehyde (TC) on the adhesion of methicillin-resistant *Staphylococcus aureus* (MRSA) cells from biofilm to host plasma and extracellular matrix proteins were evaluated. Adhesion to fibrinogen (**A**), elastin (**B**), laminin (**C**), and collagen (**D**) was assessed in the presence of TC at one-half minimum biofilm inhibitory concentration (½ MBIC) using the resazurin microtiter-plate assay. Absorbance was measured at 492 nm. Each assay was conducted in triplicate, and results were averaged. An asterisk (*) indicates that adhesion to fibrinogen, elastin, laminin, and collagen by MRSA isolates in the presence of TC was significantly reduced (*p* < 0.05) compared to the control. Adhesion inhibition (%) was determined by comparing the optical density (OD) at 492 nm of MRSA cells treated with TC at ½ MBIC to that of the untreated control.

Adhesion of MRSA isolates treated with TC at ½ MBIC to human proteins: plasma fibrinogen, fibroblast laminin, skin elastin, and type I collagen was investigated. Bacterial cells of two isolates from the wound (27887, 30216) and one isolate from the anus (1037) obtained from biofilm formed in the presence of TC, showed a reduced ability to adhere to fibrinogen compared to cells from untreated biofilm. The degree of adhesion of bacterial cells previously treated with TC to fibrinogen was significantly lower for each isolate than in the control (Table S1). Depending on the isolate, inhibition of adhesion to fibrinogen ranged from 26.1% to 41.3% (Figure 1A). Adhesion of untreated TC cells to elastin was similar to adhesion to fibrinogen (absorbance at 492 nm ranged from 1.06 to 1.1). Similarly, TC reduced cell adhesion to elastin compared with the control group, except for isolate 30216, in which no significant difference was observed between control and TC-treated cells (Table S1). Adhesion inhibition varied between isolates, ranging from 18.2% to 34.9% (Figure 1B). The adhesion of untreated TC cells to laminin and collagen was lower than to fibrinogen and elastin (absorbance values at 492 nm ranged from 0.58 to 0.68). Exposure of the biofilm cells to TC resulted in a significant reduction of adhesion to laminin and collagen. Absorbance values (OD) at 492 nm were significantly lower than those in the control group, ranging from 0.45 to 0.48. The degree of adhesion inhibition varied between isolates, ranging from 26.5% to 32.4% for laminin and from 17.1% to 30.5% for collagen (Figure 1C,D and Table S1).

### 3.2. Inhibitory Effect of TC on Enzyme Production by MRSA Isolates

**Figure 2:**
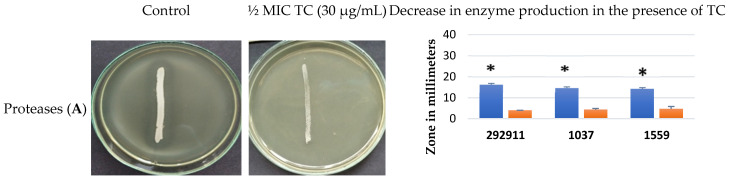

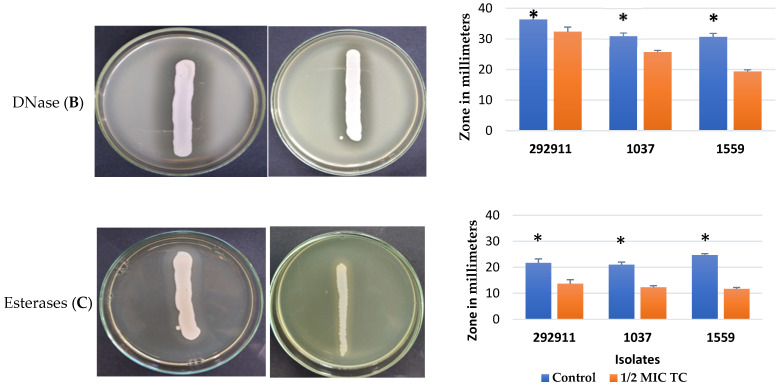
Effect of *trans*-cinnamaldehyde on the production of enzymes responsible for the virulence of MRSA isolates. (**A**)—Effect of TC on protease production by MRSA isolate (representative photo showing clear zone around MRSA isolate growth on Tryptic-Soy Agar (TSA) supplemented with 3% (*w*/*v*) bovine gelatin (control) and significantly reduced zone around isolate grown on TSA supplemented with 3% (*w*/*v*) bovine gelatin and TC (½ MIC). (**B**)—Effect of TC on DNase production by MRSA isolate (representative photo showing clear zone around MRSA isolate grown on DNase Test Lab-Agar (control) and significantly reduced zone around isolate grown on DNase Test Lab-Agar supplemented with TC (½ MIC). (**C**)—Effect of TC on esterase production by MRSA isolate (representative photo showing turbidity zones formed around the isolates growing on TSA containing 0.1% Tween 80 (control) and a significantly reduced turbidity zone around the isolate grown on this medium with TC (½ MIC). The (*) mark indicates that the production of proteases, DNase, and esterases by MRSA isolates in the presence of TC was significantly lower at *p* < 0.01 compared to control.

TC at ½ MIC reduced the production of enzymes that are important virulence factors of *S. aureus*. Protease production by MRSA isolates from the nose (292911), anus (1037), and wound (1559) was reduced at least threefold in the presence of TC compared to the untreated isolates (Figure 2A and Table S2). The clear zones containing a fraction of soluble nucleotides from degraded DNA around the isolates grown on DNase Test Lab-Agar were significantly lower in the presence of TC than on the control medium, which indicates that DNase production was significantly limited (Figure 2B and Table S3). TC also reduced esterase production by MRSA isolates because turbidity zones formed around the growing isolates containing fatty acids released by esterases and calcium ions present in the medium were significantly smaller in the TC-treated samples compared to the control (Figure 2C and Table S4).

### 3.3. Inhibitory Effect of TC on Hemolytic Activity of MRSA Isolates

**Figure 3:**
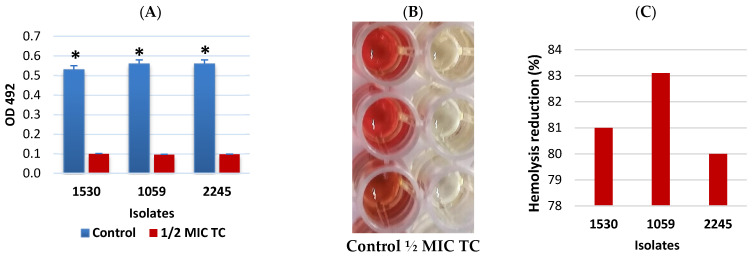
Anti-hemolytic activity of *trans*-cinnamaldehyde against MRSA isolates. (**A**)—Hemolysis of sheep red blood cells by supernatants obtained from culture of MRSA isolates incubated without (control) and in the presence of TC at ½ MIC (30 µg/mL) determined by measuring the optical density (OD) at 492 nm following 2 h incubation at 37 °C. The (*) mark indicates that the hemolytic activity of MRSA isolates in the presence of TC was significantly lower at *p* = 0.000291 compared to the control. (**B**)—Microplate wells with hemolysis caused by untreated MRSA isolates (control) and hemolysis reduction after TC treatment (½ MIC). (**C**)—Percentage of hemolysis inhibition in MRSA isolates treated with TC at ½ MIC.

Sheep erythrocytes were exposed to supernatants from MRSA isolate cultures incubated with TC at ½ MIC (30 µg/mL) to evaluate the impact of TC on MRSA hemolytic activity. The hemolytic activity of all MRSA isolates in the presence of TC was significantly reduced compared to the control group, which consisted of isolates incubated without TC (*p* = 0.000291) (Figure 3A,B). The reduction in hemolysis in the presence of TC was 80%, 81%, and 83.1% across isolates (Figure 3C and Table S5).

### 3.4. The Effect of TC on Fibroblast Viability In Vitro

**Figure 4:**
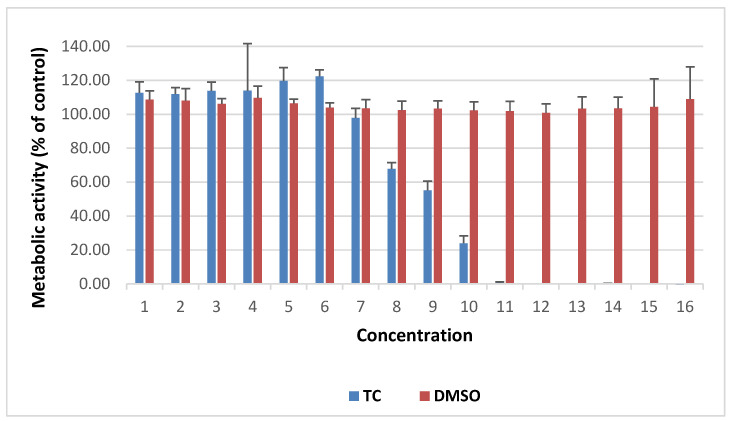
Effect of TC and DMSO on the metabolic activity of human fibroblast cells from the skin. The results of the metabolic activity of cells treated with TC and DMSO were expressed as a percentage of the control (metabolic activity of fibroblast cells in pure culture medium). Concentrations of TC and DMSO in µg/mL: **1**—0.469; **2**—0.937; **3** –1.875; **4**—3.75; **5**—7.5; **6**—15; **7**—30; **8** –60; **9** –120; **10**—240; **11**—480; **12** –960; **13**—1920; **14**—3840; **15**—7680; **16**—15360.

**Figure 5:**
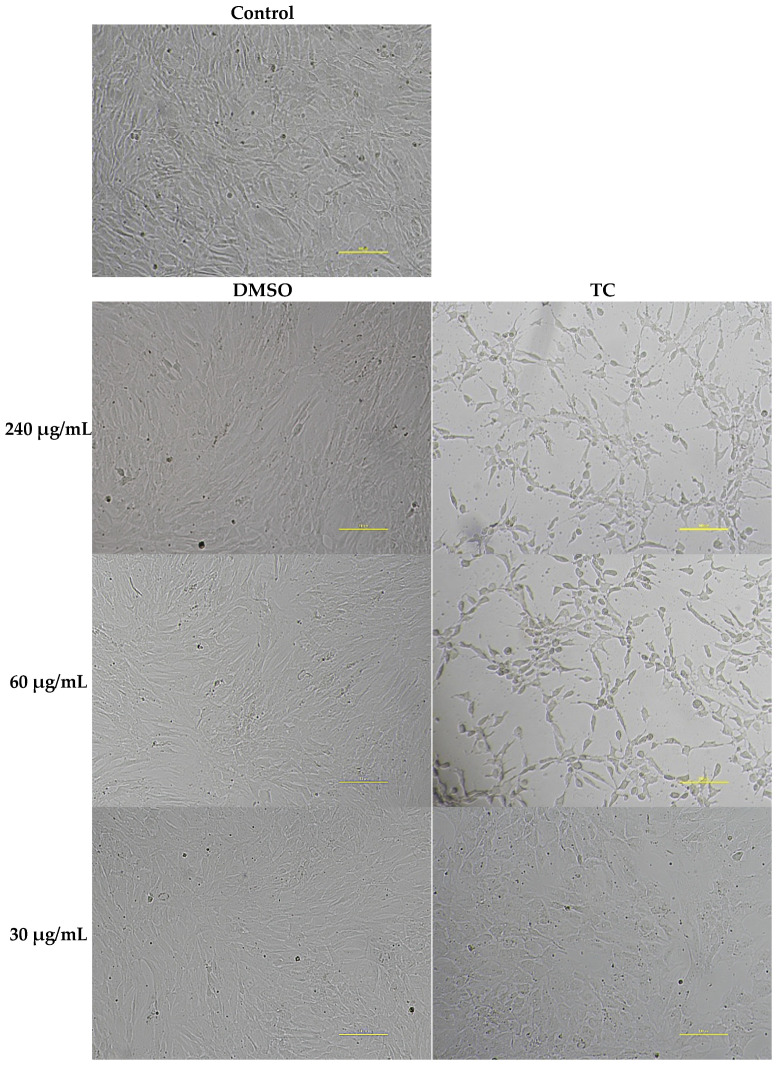
Human fibroblast cells, control (without TC and DMSO), and after 24 h of exposure to DMSO or TC at concentrations of 240, 60 and 30 µg/mL. Microscopic image at 10× magnification (Nikon Ts2R-FL, Tokyo, Japan). Scale bar = 100 µm.

TC concentration of 30 µg/mL (½ MIC) showed no cytotoxicity towards fibroblasts, with cell survival rates of 97.8% compared to the control (Figure 4 and Figure 5). However, TC at the concentrations of ½ MBIC (240 µg/mL for isolate 27887 and 60 µg/mL for isolates 1037 and 30216) showed toxic effects. At TC concentration of 60 µg/mL, 67.8% of fibroblast cells survived, while at 240 µg/mL, only 23.9% (Figure 4 and Figure 5). DMSO at the concentrations used did not show cytotoxicity against tested human fibroblasts (Figure 4 and Figure 5).

## 4. Discussion

Limiting research to the assessment of bacterial pathogen survival in the presence of antimicrobial agents is a one-way strategy, insufficient in the face of increasing drug resistance. Strategies to reduce the virulence of bacterial pathogens by inhibiting the synthesis of virulence factors necessary for bacterial survival in the host and inducing disease appear justified. Reducing the virulence potential of bacteria not only decreases the possibility of pathogenic changes in host tissues but also increases the sensitivity of bacterial cells to the immune system [18,19].

The MRSA infection process involves numerous virulence determinants, including adhesins, degradative enzymes, and toxins.

This study analysed the effect of TC on the production of selected *S. aureus* virulence factors. Attachment of *S. aureus* cells to host tissues represents the initial step in the development of staphylococcal infection. Surface proteins known as MSCRAMMs (microbial surface components recognizing adhesive matrix molecules) produced by *S. aureus* enable these bacteria to bind to various host extracellular matrix factors, including laminin, elastin, collagen, and fibrinogen present in host plasma [20,21]. Bacterial adhesion to extracellular matrix (ECM) proteins constitutes a critical step in pathogenic invasion and infection [22].

The results demonstrated that TC at ½ MBIC significantly reduced adhesion of MRSA isolates to fibrinogen from human plasma, laminin from human fibroblasts, elastin from human skin, and human type I collagen. These findings are consistent with previous studies, which reported that TC at ½ MBIC reduced the expression levels of genes encoding laminin-binding protein (*eno*), elastin-binding protein (*ebps*), and fibrinogen-binding protein (*fib*) [13].

Fibrinogen is a glycoprotein present in blood and is one of the primary proteins deposited on implanted biomaterials. The fibrinogen-binding protein serves as a crucial adherence factor, enabling *S. aureus* to attach to fibrinogen adsorbed on biomaterials and endothelial cells. Adhesion of *S. aureus* to fibrinogen induces platelet aggregation and clotting at injury sites, which can result in wound infection, colonization of implanted biomaterials, and endocarditis [23]. The present study demonstrated that, depending on the MRSA isolate, TC inhibition of adhesion to fibrinogen ranged from 26.1% to 41.3%, indicating a significantly reduced ability of isolates to adhere to this blood plasma protein and the associated pathological consequences. Elastin, a component of the extracellular matrix (ECM), facilitates *S. aureus* colonization of mammalian tissues [24]. The findings also indicated that TC significantly reduced MRSA cell adhesion to elastin compared with the control group, with reductions ranging from 18% to approximately 35% across isolates. Laminin is a major component of the blood vessel basal membrane. Binding to laminin allows staphylococcal cells to adhere to the vessel walls and disseminate through the blood, initiating tissue colonization at multiple sites of the host [25]. Collagen is the most abundant protein in mammals, primarily found in connective tissues, and numerous bacteria have evolved collagen adhesins. While collagen provides structural support for tissues, it becomes accessible to adhering bacterial cells in wounded tissues [26]. Adhesion of *S. aureus* to collagen-rich tissues facilitates infection and initiates biofilm formation [22]. The present study demonstrated that TC significantly reduced MRSA adhesion to laminin and collagen. Additionally, Yin et al. [27] reported that cinnamaldehyde reduces *Salmonella* Typhimurium’s ability to adhere to host cells by inhibiting type I fimbrial expression.

Enzymes produced by *S. aureus* that degrade host tissue components include proteases, lipases, and nucleases. *S. aureus* produces 12 extracellular proteases with unique substrate specificities and mechanisms that contribute to pathogenesis. Proteases cleave host proteins and transform bacterial cells from an adhesive to an invasive phenotype, enabling bacteria to penetrate and spread within host tissues.

Bacterial pathogens use proteases not only to facilitate invasion but also to persist in the host environment, including nutrient acquisition, bacterial dissemination, and immune evasion [28]. Our results showed that TC at ½ MIC significantly reduced protease secretion by each of the tested isolates, about 3-fold compared to the control. Other authors also showed that TC inhibited the secretion of extracellular proteases by various bacterial species. Faleye et al. [29] showed that TC slightly suppressed protease in *V. parahaemolyticus*, while derivatives of TC completely inhibited protease production at concentrations of 50 and 100 µg/mL. The inhibitory effect of TC on extracellular protease production by *Pseudomonas fluorescens* and *Enterococcus faecalis*, respectively, was also demonstrated by Li et al. [30] and Ali et al. [31].

The effect of TC on lipase production by MRSA has not been previously investigated. Lipases (glycerol ester hydrolases) are proteins associated with *S. aureus* virulence and exert detrimental effects on the host in conjunction with other bacterial enzymes, particularly phospholipases. *S. aureus* lipases significantly impair the function of various cell types involved in the human immune response, including macrophages and platelets [32]. Purified lipases induce granulocyte aggregation and reduce phagocytosis [33]. *S. aureus* cells responsible for deep infections, such as sepsis, exhibit significantly higher lipase activity than those from superficial, abscess-forming infections [34]. Mutation of the lipase gene reduces peritoneal abscess and biofilm formation in *S. aureus* [35], indicating that lipases facilitate *S. aureus* survival in biofilms and abscesses. Given the role of lipases in the pathogenesis of staphylococcal infections, identifying factors that limit lipase production is essential. The present study demonstrated that TC at ½ MIC significantly decreased esterase secretion by the investigated MRSA isolates. Li et al. [36] examined the effect of cinnamaldehyde on the virulence of *Aeromonas hydrophila* and found that 16 µg/mL of this phytochemical reduced lipase production to 73.49 ± 5.91% of the control value.

Genome sequencing of *S. aureus* revealed two staphylococcal nuclease genes, *nuc* and *nuc2* [37]. Nuc regulates biofilm formation and activates reactions that lead to apoptosis of macrophages surrounding the abscess, thereby promoting *S. aureus* survival [38,39]. Staphylococcal nuclease is a key drug target because it degrades the neutrophil extracellular traps (NET) and empowers staphylococcal cells to subvert the host’s innate immune system. The use of factors that block this activity restores effective entrapment of MRSA in DNA scaffolds. The trapped bacteria are much more easily recognized and engulfed by macrophages. Our study showed that TC at ½ MIC also significantly reduced the DNase production.

*S. aureus* produces an α-toxin that hemolyzes sheep red blood cells through partial incorporation into the target lipid bilayer and the formation of small transmembrane channels [40]. Staphylococcal α-toxin is recognized as a major virulence factor, responsible for the lysis of various cell types, including erythrocytes, platelets, epithelial cells, endothelial cells, and leukocytes [41]. The present study demonstrated that ½ MIC of TC significantly reduced the hemolytic activity of MRSA isolates, consistent with previous findings [12]. The anti-hemolytic effect of cinnamaldehyde has also been demonstrated in studies on *Streptococcus suis* type 2 [42] and *E. faecalis* [31].

These results are promising, as TC effectively blocks key virulence factors of MRSA, reducing the bacteria’s ability to spread within the host and allowing the immune response to initiate elimination of less pathogenic bacteria.

However, our studies have shown that higher doses (240 µg/mL) of TC may induce cytotoxicity in human skin fibroblast cells. Other reports indicate that TC has both genotoxic and irritant effects, although these occur at high concentrations/doses such as >500 mg/kg (systemically) or >3% (topically applied to the skin) [43]. In our study, at lower TC doses (30 and 60 µg/mL), fibroblast viability was 97.8 and 67.8%, respectively. Our results showed that topical TC use on MRSA-infected wounds may be limited by its cytotoxicity toward human skin fibroblasts at higher concentrations. However, in our study, for some MRSA isolates, lower concentrations of TC were sufficient to limit the production of virulence factors, while fibroblast viability remained high, indicating a lack of cytotoxicity, according to ISO 10993-5 (2009) [44]. Figueiredo et al. [18] investigated the effects of topical administration of TC (200 µg/wound/day) on experimental wounds in Swiss mice infected with *S. aureus*. TC treatment enhanced healing and reduced the severity of *S. aureus*-infected skin lesions, correlating with a significant reduction in bacterial load in TC-treated wounds.

Cinnamaldehyde is regarded as safe and well-tolerated in both humans and animals [45,46]. The Food and Drug Administration (FDA) and the Council of Europe recommend an acceptable daily intake of 1.25 mg/kg. Nevertheless, additional studies are required to establish the safety and efficacy of orally administered cinnamaldehyde in humans.

Overall, we suggest that TC may represent an alternative therapy for combating bacterial infections caused by *S. aureus*, as it demonstrates the ability to reduce bacterial virulence and survival, thereby improving the host’s immune response to infection. We demonstrated that TC has antimicrobial activity that disrupts *S. aureus* virulence, which is important for successful wound healing. However, exploring the synergistic effect of TC with other antibacterials, which would enable its dose to be reduced, would make it possible to use TC in topical formulations (aerosol formulations, gel, nanoemulsion) for the treatment of skin infections caused by *S. aureus*.

Future research should include a larger number of MRSA isolates to gain broader insight into the influence of TC on MRSA virulence factors, and the genome sequences of these isolates should be analyzed.

In addition to assessing the effect of TC on the expression of adhesion-related genes, which have been assessed previously [13], the effect of TC on the expression of genes related to protease, lipase, and DNase production, as well as the *hla* gene encoding alpha-hemolysin, should be investigated in the future. Furthermore, studies using other cell lines would provide greater knowledge of TC cytotoxicity and safe concentrations for the host.

## 5. Conclusions

Reducing the virulence potential of bacteria not only reduces the possibility of pathological changes in host tissues but also increases the sensitivity of bacterial cells to the immune system.

The inhibitory effect of TC on *S. aureus* adhesion to host proteins such as fibrinogen, elastin, laminin, and collagen prevents the dissemination of staphylococcal cells and the initiation of host tissue colonization.

Inhibition of MRSA protease, DNase and lipase production, and reduction of MRSA-induced hemolysis may limit tissue damage and inflammatory processes during infection.

Based on these findings, we believe that TC may be an alternative to antibiotics used to treat MRSA-induced infections. However, the toxicity of high concentrations of TC to human fibroblasts raises the need for further research on the synergistic effect of TC with other antibacterials, which would enable its dose to be reduced.

## Figures and Tables

**Table 1:** The MRSA isolates used in study.

Isolate ID	Source of Isolation	TC Concentrations (µg/mL)
Adhesion assay		
1037	Anus	60
27887	Wound	240
30216	Wound	60
Assay of protease, DNase and esterase production		
292911	Nose	30
1037	Anus	30
1559	Wound	30
Hemolysis assay		
1530	Wound	30
1059	Nose	30
2245	Wound	30

## Data Availability

All original contributions presented in this study are included within the article. Additional information is available from the corresponding author upon request.

## Supplementary Materials

The following supporting information can be downloaded at: https://www.mdpi.com/article/10.3390/pathogens15030271/s1, Table S1. Effects of *trans*-cinnamaldehyde (TC) on adhesion of MRSA cells from biofilm to proteins present in the host plasma and extracellular matrix; Table S2. Effect of *trans*-cinnamaldehyde on the production of proteases by MRSA isolates; Table S3. Effect of *trans*-cinnamaldehyde on the production of DNase by MRSA isolates; Table S4. Effect of *trans*-cinnamaldehyde on the production of esterases by MRSA isolates; Table S5. Effect of *trans*-cinnamaldehyde on hemolytic activity of MRSA isolates.
