# Supplementary material for: Effect of trans-Cinnamaldehyde on Adhesion and Other Virulence Factors of Methicillin-Resistant Staphylococcus aureus
Source: Pathogens. 2026 Mar 3;15(3):271. doi: 10.3390/pathogens15030271 (PMC13028670; doi:10.3390/pathogens15030271)
Supplement: Supplementary file 1 [file pathogens-15-00271-s001.zip › pathogens-4151146-supplementary.pdf]

Supplementary Materials

Table S1. Effects of *trans*-cinnamaldehyde (TC) on adhesion of MRSA cells from biofilm to proteins present in the host plasma and extracellular matrix.

Isolates	Source of isolation	Control	TC at ½ MBIC	P-value	Adhesion inhibition (%)
		OD_{492} (mean ± SD)			
		Adhesion to fibrinogen			
1037	Anus	1.074±0.100	0.794 ±0.039	0.000247	26.1
27887	Wound	1.027±0.049	0.737±0.060	0.000188	28.4
30216	Wound	1.151±0.115	0.675±0.045	0.000187	41.3
		Adhesion to elastin			
1037	Anus	1.073±0.064	0.877±0.078	0.000915	18.2
27887	Wound	1.063±0.073	0.691±0.067	0.000188	34.9
30216	Wound	1.106±0.073	0.744±0.040	0.320	32.7
		Adhesion to laminin			
1037	Anus	0.665±0.026	0.451±0.010	0.000187	32.1
27887	Wound	0.633±0.015	0.466±0.012	0.000187	26.5
30216	Wound	0.670±0.012	0.474±0.002	0.000187	29.4
		Adhesion to collagen			
1037	Anus	0.578±0.025	0.484±0.007	0.000189	17.1
27887	Wound	0.680±0.026	0.472±0.003	0.000187	30.5
30216	Wound	0.679±0.021	0.472±0.008	0.000187	30.3

Table S2. Effect of *trans*-cinnamaldehyde on the production of proteases by MRSA isolates.

Isolates	Source of isolation	Control	TC at ½ MIC (30 μg/mL)	P-value
		Clear zone in millimetres (mean ± SD)		
292911	Nose	16.33±0.57	4.00	0.000291
1037	Anus	14.67± 0.57	4.33±0.57	0.000296
1559	Wound	14.33±0.57	4.67±1.15	0.000434

Table S3. Effect of *trans*-cinnamaldehyde on the production of DNase by MRSA isolates.

Isolates	Source of isolation	Control	TC at ½ MIC (30 μg/mL)	P-value
		Clear zone in millimetres (mean ± SD)		
292911	Nose	36.33±1.52	32.33±1.52	0.03286
1037	Anus	31.00±1.0	25.67±0.57	0.001538
1559	Wound	30.67±1.15	19.33±0.57	0.000358

Table S4. Effect of *trans*-cinnamaldehyde on the production of esterases by MRSA isolates.

Isolates	Source of isolation	Control	TC at ½ MIC (30 μg/mL)	P-value
		Clear zone in millimetres (mean ± SD)		
292911	Nose	21.67±1.52	13.67±1.52	0.003231
1037	Anus	21.00±1.0	12.33±0.57	0.000433
1559	Wound	24.67±1.15	11.67±0.57	0.000292

Table S5. Effect of *trans*-cinnamaldehyde on hemolytic activity of MRSA isolates.

Isolates	Source of isolation	Control	TC at ½ MIC (30 μg/mL)	P-value	Hemolysis reduction (%)
			OD_{492}		
1530	Wound	0.53±0.017	0.1±0.002	0.000291	81
1059	Nose	0.56±0.016	0.1±0.002	0.000291	83.1
2245	Wound	0.56±0.022	0.1±0.001	0.000291	80
